# Spatial and Temporal Variations of Ecosystem Service Values in Relation to Land Use Pattern in the Loess Plateau of China at Town Scale

**DOI:** 10.1371/journal.pone.0110745

**Published:** 2014-10-20

**Authors:** Xuan Fang, Guoan Tang, Bicheng Li, Ruiming Han

**Affiliations:** 1 Key Laboratory of Virtual Geographic Environment, Ministry of Education, School of Geography Science, Nanjing Normal University, Nanjing, China; 2 Research Center of Soil and Water Conservation and Ecological Environment, Chinese Academy of Sciences, Yangling, Shaanxi, China; 3 School of Geography Science, Nanjing Normal University, Nanjing, China; University of Brasilia, Brazil

## Abstract

Understanding the relationship between land use change and ecosystem service values (ESVs) is the key for improving ecosystem health and sustainability. This study estimated the spatial and temporal variations of ESVs at town scale in relation to land use change in the Loess Plateau which is characterized by its environmental vulnerability, then analyzed and discussed the relationship between ESVs and land use pattern. The result showed that ESVs increased with land use change from 1982 to 2008. The total ESVs increased by 16.17% from US$ 6.315 million at 1982 to US$ 7.336 million at 2002 before the start of the Grain to Green project, while increased significantly thereafter by 67.61% to US$ 11.275 million at 2008 along with the project progressed. Areas with high ESVs appeared mainly in the center and the east where largely distributing orchard and forestland, while those with low ESVs occurred mainly in the north and the south where largely distributing cropland. Correlation and regression analysis showed that land use pattern was significantly positively related with ESVs. The proportion of forestland had a positive effect on ESVs, however, that of cropland had a negative effect. Diversification, fragmentation and interspersion of landscape positively affected ESVs, while land use intensity showed a negative effect. It is concluded that continuing the Grain to Green project and encouraging diversified agriculture benefit to improve the ecosystem service.

## Introduction

Ecosystem contributes to human welfare by providing goods and services directly and indirectly [1]–[2]. With widely spreading of environmental problems, ecosystem service received increasing attention. Many studies showed human factors, such as urban sprawl [3], [4], [5], socioeconomic changes [6], agricultural policies [7], [8], could affect natural or artificial ecosystems. Land use, an original and foundational human activity and represents the most substantial human alteration to systems on the planet of earth for long-term study [9], plays an important role in providing ecosystem services, including biodiversity, water filtration, retention of soil, etc. [10] Inappropriate land use may lead to significant degradation of local and regional ecological services [11]. Moreover, there were studies showed that ecosystem service trade-offs could successful apply to land use planning [12], [13]. Understanding the relationship between ecosystem services and land use change is essential for maintaining a healthy ecosystem and getting sustainable services.

The growing body of literatures focused on how ecosystem service changes in response to land use change of different regions [14], [15], [16], [17], [18]. However, these studied focus on the impact of land use type on ecosystem service, while the spatial pattern of land that reflects ecological processed and functions [19] get less attention. Monitoring the characteristic of landscape patterns including area, shape, diversity, etc., is helpful to deeply understand the relationship between ecosystem service and land use change and then to provide complete references for land use planning.

The Loess Plateau is the area suffered from the most severe soil erosion in the world, and it is also a major agricultural production region in China [20]. Long-term poor land use has resulted in vegetation destruction and accelerated soil erosion [21]. To control soil erosion and restore the ecosystem, the Grain for Green project converting slope cropland to grassland or forestland was implemented in 1999 by the Chinese Government [22]. The land use on the plateau under the project has changed significantly. Studying the ecosystem service in relation to land use change before and after the Grain to Green project was crucial for ecosystem protection and agricultural sustainability for the area. Researchers have analyzed ecosystem service at different scales within the Loess Plateau [17], [18], [23]. However, town is a basic administrative area in China. Exploring the characteristic of ecosystem services change at town scale is of practical significance to provide operable land use planning.

Ecosystem service values (ESVs) is monetary assessment of ecosystem services. This paper examined the characteristics of ESVs at Hechuan town, a typical town in the hilly and gully region of the Loess Plateau. The objectives of this study were: 1) to analyze the changes in land use pattern from 1982 to 2008; 2) to access the spatial and temporal variation in ESVs in response to land use during this period; 3) to quantitively analysis the relationship between ESVs and land use pattern; and 4) to discuss how land use management is favorable for ecosystem service supply and the ecological and economic sustainable development.

## Data and Methods

### 2.1 Ethics statement

No specific permits were required for the described studies, and the work did not involve any endangered or protected species.

### 2.2 Study area

**Figure 1:**
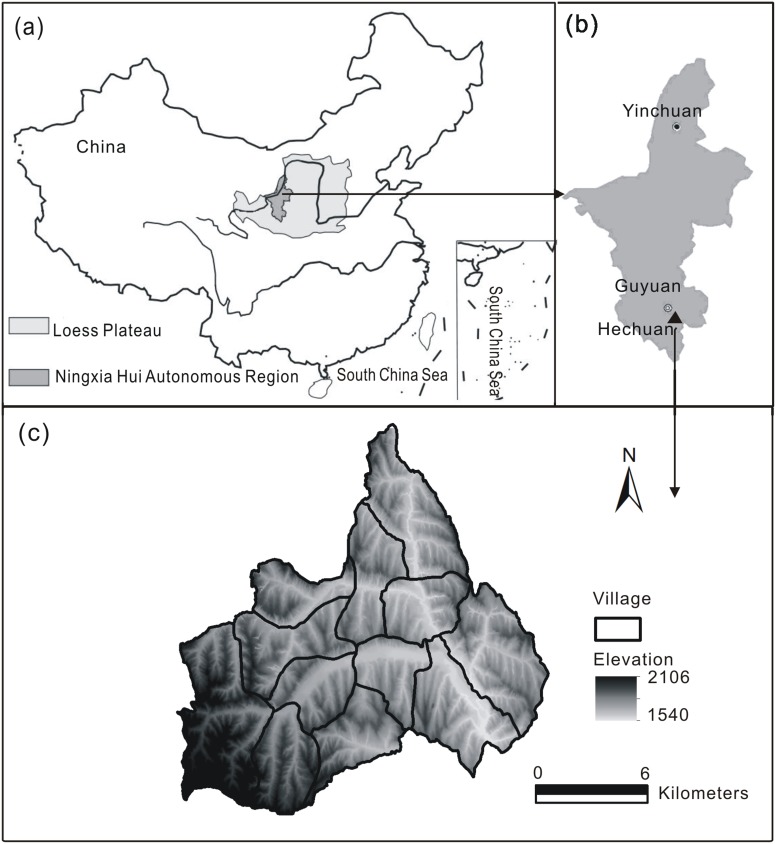
Location of the study area. Ningxia Province and the Loess Plateau, China (a), the location of Hechuan Town in the Loess area of Ningxia Province (b) and, the village boundary and the digital elevation model (DEM) map of Hechuan Town (c).

The study area, Hechuan town (106°18′43″∼106°32′16″E, 35°54′59″∼36°06′05″N), is located in Guyuan city of the Ningxia Hui Autonomous Region of northwest China (Fig. 1), consisting 12 villages with 16,524 people. The reasons that Hechuan Town was chosen as the study area were, on the one hand, Hechuan town has the typical characteristics of Loess Plateau including the terrain of hill and gull, the fragile ecosystem and the backward economy; on the other hand, there was a long term ecological observation and experiment station in the study area, which facilitated the survey of land use and ecosystems. This town has an altitude ranging from 1540 to 2106 m, covering an area of 215.58 km^2^. There exist the topographic differences in the town. The central area with river terrace stretches smoothly with a low elevation. The terrain in the northern area is fragmented while that of southern area is relatively simple. Hechuan town has a semi-arid continental temperate climate with the average annual temperature of 6.9°C and precipitation of 419 mm (1982–2002). Most of the annual precipitation is concentrated between June to September in the form of heavy storms that can cause severe soil erosion. The soil is composed of loessial soil and Dark loessial soils, which is erodible due to its weak cohesion and high infiltrability.

The ecosystem in Hechuan town is fragile with serious soil erosion and frequent natural disasters. Human disturbances of excessive land use, such as deforestation, overgrazing and over-reclamation further destructed the native natural grassland. Therefore, this area has long been in a vicious circle, endless cultivation and poverty. Since the early 1980s, a variety of comprehensive investigation of soil erosion was practiced by Chinese Academy of Sciences. Shanghuang watershed, located in the east of Hechuan town, was taken as a key test area. The ecological restoration covering the whole town was started from implementing the Grain for Green project after 2002 (launched in 1999 by China government). Since then, abandoned cropland, shrubland (*Caragana korshinskii*, *Hippophae rhamnoides*) and artificial grassland (*Medicago sativa*) was generated, which made a significant change on landscape pattern and ecosystem components providing a variety of ecosystem services. Meanwhile, farming and grazing, the traditional way of living, had to be changed, and raising livestock, orchards, and migrant working diversified their incomes.

### 2.3 Data acquisition and preprocessing

**Figure 2:**
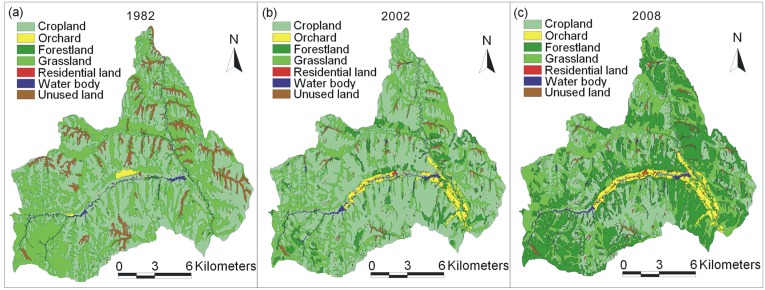
Land use maps of Hechuan town in 1982 (a), 2002 (b) and 2008 (c).

Land use data was the key data for evaluating landscape pattern and ecosystem service. The land use data of 1982 was obtained by digitizing the land use patches from the 1∶10,000 scale topographic maps of 1982, in which the information of land use types and its boundary are clearly shown. The 10 m resolution of remote sensing image could be considered to be corresponding with the scale of 1∶50000 [24], [25]. The land use data of 1982 acquired from 1∶10000 topographic maps was therefore generalized to be at 1∶50000 scale [26]. The land use data of 2002 and 2008 were respectively extracted from the 10 m resolution multispectral Spot-5 image of 2002 and 2008 by updating the land use patches of 1982 one by one in visual interpretation method. The interpretation sign was established by understanding the Spot image characteristics and carrying out field surveys in order to further determine the relationship between the true ground and the image. The kappa accuracy index [27] was used to assess the accuracy of the interpretation. The stratified random sampling method was used to generate the reference points on the classified image for the accuracy test. These reference points were located in the field with a GPS with 5-m precision for ground truth. The total kappa indexes are all higher than 0.85, which are higher than the minimum acceptable (0.7) [28]. Considering the characteristic of the land use in study area and the interpretation level of the data and to facilitate the calculation of ESVs, the land use was classified into seven types: cropland, orchard, forestland, grassland, residential area, water area, and unused land (Fig. 2).

To acquire accurate area data of the land use for ESVs estimation and facilitate analyzing the spatial distribution of ESVs, the topographic maps and Spot images were transformed to the same projection and coordinate system (the Albers-Conical-Equal-Area projection system and Krasovsky 1940 coordinate system) before the extraction of land use data, and all acquired land use data were transformed to Arc-grid formats with the same grid size (10 m×10 m). The above data processing was completed using ERDAS and ArcGIS software.

### 2.4 Analysis on land use pattern

The transfer matrix analysis of land use was produced to understand how land use changed. Landscape metrics analysis was used for spatial pattern analysis of land use. Landscape metrics has been adopted widely; meanwhile, its abilities to indicate ecological process gained increasing attention [29], [30], [31]. Conceptual flaws in landscape pattern analysis, limitations inherent in landscape metrics and the improper use of pattern analysis may lead to the misuse of landscape metrics [32]. For better explanations and predictions of ecological phenomena from ecological pattern, the landscape metrics in this study was therefore selected by two steps. Firstly, the diversity, the fragmentation and the dominance of landscape were all considered, and then 34 metrics was selected, by understanding the knowledge of the landscape pattern and the ecological services indication of landscape metrics [33], [34] and referring to the previous studies on landscape pattern [4], [31], [35], [36]. Secondly, a correlation analysis for the 34 metrics was employed to ensure the low redundancy among landscape metrics. If the coefficient between two metrics was significant at 0.05 level, only one metric of them could be eventually selected.

Landscape-level metrics providing general landscape information and class-level metrics providing more specific information about variations at the local level and spatial patterns of land use classes [37] were used to monitor the characteristics of landscape pattern. The selected landscape-level metrics were patch density (PD), area-weighted mean shape index (SHAPE_AM), Interspersion and Justaposition Index (IJI), and Shannon’s diversity index (SHDI). The selected class-level metrics were PD, the percentage of landscape (PLAND), SHAPE_AM and IJI. PD and SHAPE_AM could show the fragmentation of landscape. SHDI and PLAND reflect the dominance of some land use type and the diversity of landscape, respectively. IJI reflects whether the patches or classes are contiguous. Landscape metrics analysis was conducted with above metrics by FRAGSTATS 3.3, in which the eight-neighbor rule was used to derive the patch number. Besides these metrics, the land use intensity index (LUII) was also used to describe the landscape pattern. It was calculated by the following equation [31]:
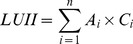
(1)where *LUII* is the land use intensity index, *A_i_* is the percentage of for a give land use type *i*, and *C_i_* is the coefficient value of intensity for a give land use type *i*, that is assigned 4 for build-ups, 3 for farmland and 2 for forest, orchard, grassland and water bodies, and 1 for unused land.

### 2.5 Estimation of ESVs

Costanza et al.’s model of ESVs estimation was adopted in this study [1], [2]. The model classified ecosystem service into 17 types of service functions and estimated the ESVs by placing an economic value on different biomes [34]. For the defects of this model, such as overestimating the agriculture ESVs and underestimating the wetland ESVs, Xie et al. proposed refined coefficients for ESVs assessment both solving the above problem and making it apply to China [33], [34]. Based on this model, the total ESVs in the study area was calculated using the following formulas:
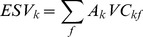
(2)

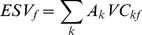
(3)

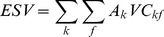
(4)where *ESV_k_*, *ESV_f_*, *ESV* are the ESVs of land use type *k*, the ESVs of ecosystem service function type *f*, and the total ESVs respectively. *A_k_* is the area (ha) for land use types. *VC_kf_* is the value coefficient (US$·ha-1·yr-1) for land use type *k* and ecosystem service function type *f*, which is the key for ESVs estimating. Xie et al.’s model was used to determine *VC_kf_*, which can be expressed as follows:
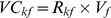
(5)where *R_kf_* is the equivalent weight factor of ecosystem service, *V_f_* is food production values of agriculture land per area per year.

The equivalent weight factor was presented for customizing Chinese terrestrial ecosystem based on Costanza et al.’s model by surveying 500 Chinese ecologists (Table 1) [34]. It is the ratio of the ESVs to the economic value of average natural food production provided by agricultural land per hectare per year. The factors of land use types in our study were basically assigned based on the nearest ecosystems in Xie et al.’s model. However, minor adjustments were made. The equivalent weight factor of orchard which was not put forward clearly in Xie et al.’s model was determined by the mean of grassland and forestland by referring some researches [5], [18]. The factor of unused land equates to that of barren land, and that of residential land was determined to zero.

**Table 1:** Equivalent weight factor of ecosystem service values (ESVs) per hectare of terrestrial ecosystem in China [30].

	Cropland	Forestland	Grass land	Water body	Barren land
Gas regulation	0.72	4.32	1.5	0.51	0.06
Climate regulation	0.97	4.07	1.56	2.06	0.13
Water supply	0.77	4.09	1.52	18.77	0.07
Soil formation and retention	1.47	4.02	2.24	0.41	0.17
Waste treatment	1.39	1.72	1.32	14.85	0.26
Biodiversity protection	1.02	4.51	1.87	3.43	0.40
Food production	1.00	0.33	0.43	0.53	0.02
Raw material	0.39	2.98	0.36	0.35	0.04
Recreation and culture	0.17	2.08	0.87	4.44	0.24
Total	7.90	28.12	11.67	45.35	1.39

The value of food production service of agriculture land per area per year was considered to be 1/7 of the actual price of food production in Xie et al.’s model. With the average actual food production of cropland in Hechuan town from 1982 to 2008 of 901.77 kg/ha which was get from *Statistic yearbook of the Yuanzhou District, Guyuan City, Ningxia Hui Autonomous Region* and the average grain price of US$ 0.243 per kilogram (i.e. an equivalent of RMB Yuan 1.69 according to the average exchange rate of 2008) in 2008, the value of food production service of cropland per area per year was calculated to be US$ 31.348 (i.e. an equivalent of RMB Yuan 217.713 according to the average exchange rate of 2008). ESVs of one unit area of each land use types were then assigned as shown in Table 2.

**Table 2:** The ecosystem service values (ESVs) per hectare of different land use types in Hechuan town (US$·ha-1·yr-1).

	Cropland	Orchard	Forestland	Grass land	Water body	Unused land
Gas regulation	22.570	91.222	135.422	47.022	15.987	1.881
Climate regulation	30.407	88.244	127.585	48.902	64.576	4.075
Water supply	24.138	87.930	128.212	47.649	588.397	2.194
Soil formation and retention	46.081	98.118	126.018	70.219	12.853	5.329
Waste treatment	43.573	47.649	53.918	41.379	465.514	8.150
Biodiversity protection	31.975	99.999	141.378	58.620	107.523	12.539
Food production	31.348	11.912	10.345	13.480	16.614	0.627
Raw material	12.226	52.351	93.416	11.285	10.972	1.254
Recreation and culture	5.329	46.238	65.203	27.272	139.184	7.523
Total	247.647	623.663	881.498	365.828	1421.619	43.573

After the ESVs were calculated by above processing, a sensitivity analysis was conducted to test the land use type’s representative for ecosystem types and the certainty of the coefficients value for ecosystem service. A coefficient of sensitivity (CS) was used to indicate the degree of sensitivity of ESVs to a coefficients value, calculated by the following formula [5]:
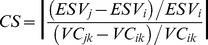
(6)where *ESV_j_* an *ESV_i_* are the total ESVs of the initial status j and the adjusted status *i*, and *VC_jk_* and *VC_ik_* are the initial and adjusted coefficients. A 50% adjustment in the coefficients was made in the study. The greater the CS responded to the adjustment, the more critical is the use of an accurate coefficient [38]. A CS lower than 1 indicates the ESVs is inelastic to the coefficient and the estimation of ESVS is reliable. Otherwise, a CS greater than 1 indicates the estimation of ESVs is sensitive to the coefficient.

### 2.6 Correlation and regression analysis

The data of ESVs and landscape metrics was used to analysis the relationship between ecosystem service and land use pattern change. Because the spatial variation of landscape pattern exist among 12 villages in Hechuan town, the land use data of the three years (1982, 2002 and 2008) for the 12 villages can be considered as representing different landscape pattern on a time-for-space perspective [39]. Therefore, there were totally 36 sample data. Correlation and regression was employed for the relationship analysis, in which Multiple stepwise regression was specifically chosen considering the multicollinearity among landscape metrics. The dependents were the nine categories and total ESVs, while the corresponding independents were the landscape-level and class-level landscape metrics.

## Results

### 3.1 Changes of land use pattern

Table 3 showed the land use transition matrix. From 1982 to 2002, cropland as the dominant land use type increased from 50.83% to 58.76%. Grassland was the land use type with the largest change in area, decreasing from 40.01% to 28.77%. Orchard increased by 6.24% of total area, indicating the economic driving force of fruit trees on land use change. Forestland increased from 0.57% to 2.78%, reflecting that ecological restoration began to gain attention. From 2002 to 2008, cropland and forestland changed significantly, decreasing from 58.76% to 27.27% and increasing from 6.51% to 34.05% respectively. Land use structure was transferred from cropland dominated (58.76%) to cultivated land (27.27%), forestland (34.05%) and grassland (31.91%) relatively balanced distributed.

**Table 3:** Land use transition matrix from 1982 to 2002 and from 2002 to 2008 (%).

	2002								
1982	Cropland	Orchard	Forestland	Grassland	Residential land	Water body	Unused land	Total	Loss
Cropland	44.42	2.33	1.18	2.50	0.35	0.04	0.01	50.83	6.41
Orchard	0.17	0.05	0.26	0.09	0.00	0.00	0.00	0.57	0.53
Forestland	0.10	0.01	0.15	0.02	0.00	0.00	0.00	0.28	0.13
Grass land	12.63	0.39	4.74	22.15	0.01	0.08	0.00	40.01	17.86
Residential land	0.05	0.01	0.00	0.01	0.14	0.00	0.00	0.22	0.07
Water body	0.01	0.00	0.01	0.00	0.00	0.80	0.00	0.81	0.02
Unused land	1.39	0.00	0.16	3.98	0.00	0.07	1.66	7.27	5.61
Total	58.76	2.78	6.51	28.77	0.51	0.99	1.68	100.00	
Gain	14.35	2.73	6.36	6.62	0.37	0.19	0.01		
	**2008**								
**2002**	**Cropland**	**Orchard**	**Forestland**	**Grass land**	**Residential land**	**Water body**	**Unused land**	**Total**	**Loss**
Cropland	27.09	1.00	20.75	9.84	0.07	0.00	0.00	58.76	31.68
Orchard	0.00	2.75	0.01	0.00	0.01	0.00	0.00	2.78	0.03
Forestland	0.05	0.04	5.84	0.57	0.01	0.01	0.00	6.51	0.67
Grass land	0.12	0.00	7.17	21.42	0.02	0.04	0.00	28.77	7.35
Residential land	0.00	0.00	0.00	0.00	0.51	0.00	0.00	0.51	0.00
Water body	0.02	0.02	0.00	0.01	0.00	0.94	0.00	0.99	0.05
Unused land	0.00	0.00	0.27	0.08	0.00	0.00	1.33	1.68	0.35
Total	27.27	3.82	34.05	31.91	0.62	1.00	1.33	100.00	
Gain	0.18	1.07	28.21	10.50	0.11	0.05	0.00		

The most notable change of land use from 1982 to 2002 was the conversion from grassland to cropland and forestland with 12.63% and 4.74% of the total area respectively. The conversions from cropland (2.50%) and unused land (3.98%) to grassland were not adequate to compensate for the grass loss. From 2002 to 2008, the notable changes of land use were cropland to forestland, cropland to grassland, and grassland to forestland, with the rates of 20.75%, 9.84%, and 7.17% respectively. It was found that the conversion among land use types was more outstanding and concentrated than that before 2002, reflecting that the Grain for Green project as an ecological policy had great influence on land use change.

**Figure 3:**
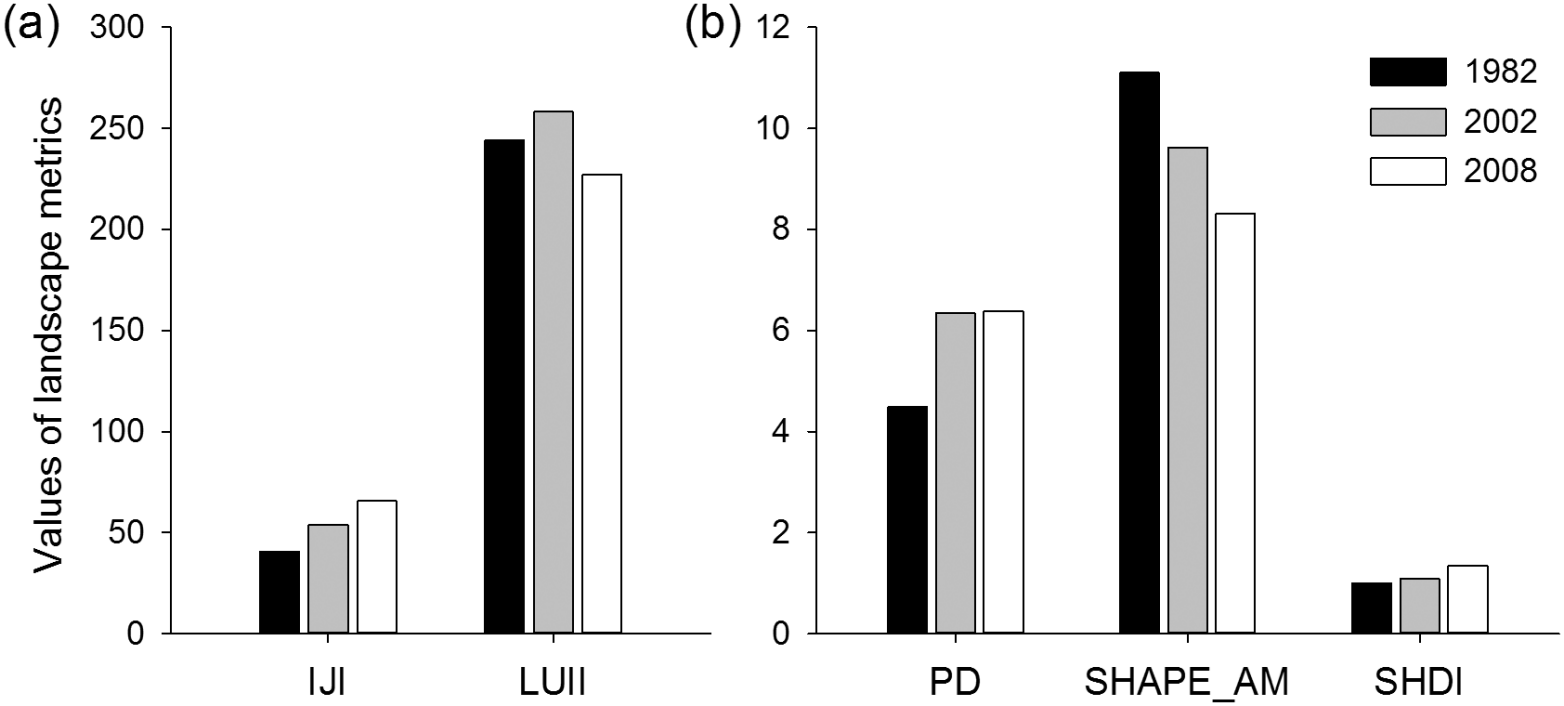
Landscape metrics at the landscape level in Hechuan Town in 1982, 2002 and 2008. IJI: Interspersion and Justaposition Index; LUII: land use intensity index; PD: patch density; SHAPE_AM: area-weighted mean shape index; SHDI: Shannon’s diversity index.

The results of landscape-level metric analysis were exhibited in Fig. 3. The significant increased PD from 1982 to 2002 reflected the landscape fragmentation. It was relative to the increase of patches on the land use types with intense human disturbance, such as cropland, residential land and artificial reservoir. Oppositely, the slight change of PD from 2002 to 2008 reflected that human disturbance became stable. The change of human disturbance was also demonstrated by the change of LUII which increased before 2002 and decreased after 2002. SHAPE_AM decreased in the study period, showing the landscape became more regular in shape. The increase of IJI suggested that the landscape became more contiguous and the ecological connectivity among land use types increased. SHDI increase obviously from 2002 to 2008, which related to that the land use structure became even.

**Figure 4:**
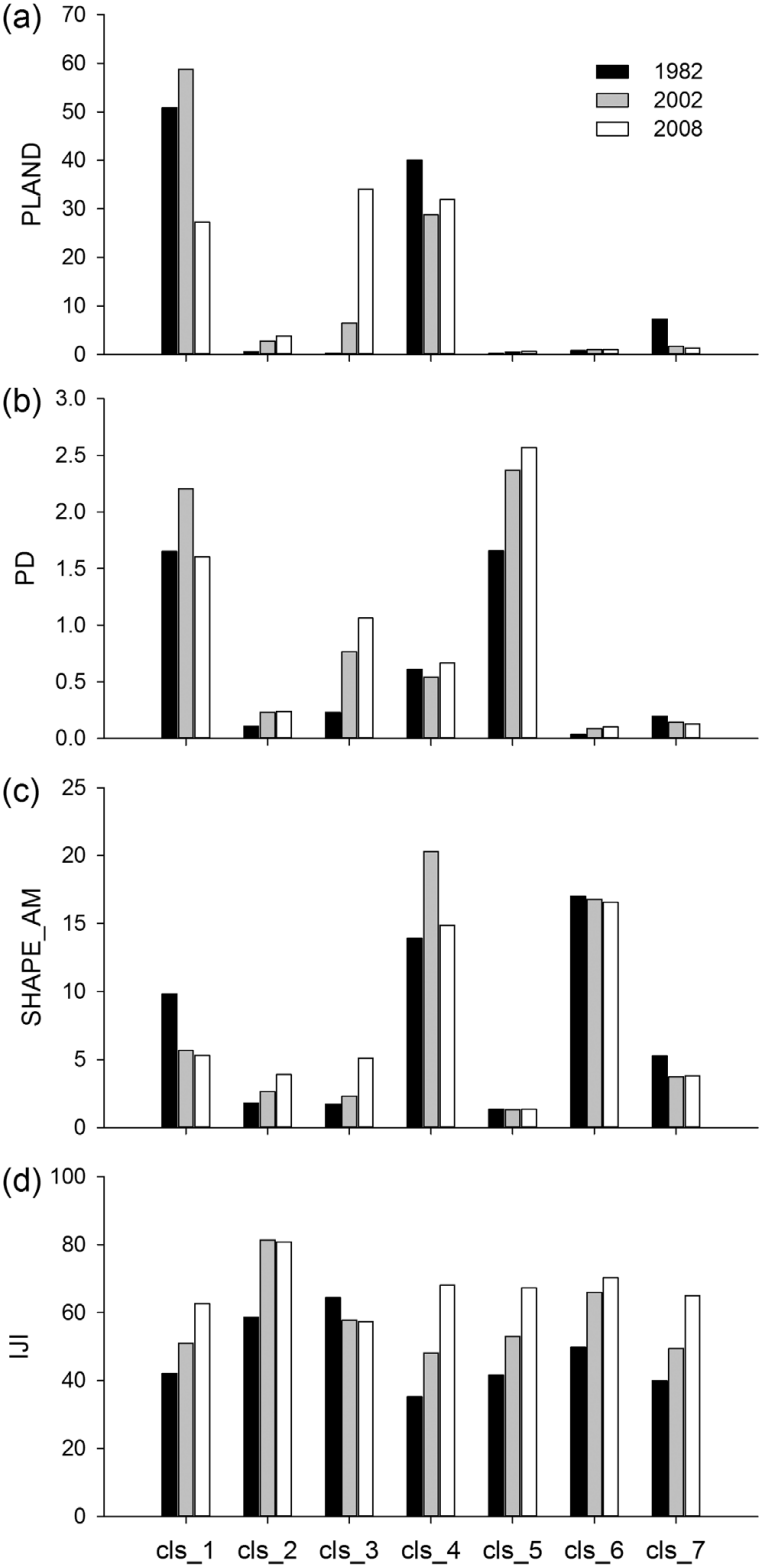
Landscape metrics at the class-level in Hechuan Town in 1982, 2002 and 2008. cls_1, cls_2, cls_3, cls_4, cls_5, cls_6, and cls_7 represent cropland, orchard, forestland, grassland, residential land, water body and unused land. PLAND: the percentage of landscape; PD: patch density; SHAPE_AM: area-weighted mean shape index; IJI: Interspersion and Justaposition Index.

Fig. 4 showed the change of class-level metrics. The PLAND of land use types indicated that cropland, forestland, and grassland had significantly influence on land use pattern. PD in orchard, forestland, and residential land increased obviously, attributing to the increasing area of these land use types and the fragmental terrain. SHAPE_AM showed that cropland and unused land became more regular in shape, while orchard and forestland more complicated. IJI increased generally in land use types. Orchard was the most contiguous with high IJI, which was relative to its concentrated distribution across the river terrace.

### 3.2 ESVs from 1982 to 2008

The ESVs of each land use type and the total ESVs was shown in Table 4. The total ESVs of Hechuan town was US$ 6.315, US$ 7.336 and US$ 11.275 million in 1982, 2002 and 2008, respectively. From 1982 to 2002, the decline of ESVs caused by the decrease of grassland was offset by the increase of forestland, orchard and cropland, resulting that the total ESVs increased by US$ 1.021 million. From 2002 to 2008, the total ESVs increased by US$ 3.939 million, mainly due to the increase of forestland. The average annual change rate of total ESVs before and after 2002 was quite different, that is 0.81% and 8.95% respectively. It indicated the Grain to Green project implemented since 2002 had a significant effect on the ecosystem service. It was also shown from the value of ESVs produced by forestland occupying 57.39% of the total ESVs. Overall, the total ESVs increased US$ 4.960 million during the study period, mainly due to the increase of ESVs by the increase of forestland and orchard far beyond the decrease of ESVs by the decrease of cropland and grassland. It was essentially because of the higher coefficient value of forestland and orchard than that of cropland and grassland.

**Table 4:** The change of ecosystem service values (ESVs) in Hechuan Town from 1982 to 2008.

		Cropland	Orchard	Forestland	Grass land	Water body	Unused land	Total
ESVs	1982	2.714	0.077	0.051	3.155	0.249	0.068	6.315
(10^6^ US$ yr^−1^)	2002	3.137	0.374	1.237	2.269	0.304	0.016	7.336
	2008	1.456	0.514	6.470	2.517	0.305	0.013	11.275
Change of ESVs	1982–2002	0.423	0.297	1.186	−0.887	0.054	−0.053	1.021
(10^6^ US$ yr^−1^)	2002–2008	−1.681	0.140	5.234	0.248	0.002	−0.003	3.939
	1982–2008	−1.258	0.437	6.419	−0.638	0.056	−0.056	4.960
Change of ESVS	1982–2002	2.248	55.387	333.431	−4.045	3.145	−11.081	2.328
(%)	2002–2008	−7.716	5.404	60.934	1.574	0.072	−2.992	7.731
	1982–2008	−6.674	81.578	1805.410	−2.913	3.232	−11.771	11.309
Average annual Change	1982–2002	0.112	2.769	16.672	−0.202	0.157	−0.554	0.117
(%yr^−1^)	2002–2008	−1.286	0.901	10.155	0.262	0.012	−0.498	1.289
	1982–2008	−0.256	3.137	69.439	−0.112	0.124	−0.452	0.435

The ESVs of each ecosystem function type was shown in Table 5. Expect for food production, the values of ecosystem service functions increased especially after 2002. The decrease of food production was due to the great decline of cropland in the Grain to Green project. The ESVs proportion of each ecosystem function type to the total ESVs represented the contribution of each ecosystem function to the total ESVs. It was found that the functions of soil formation and retention, waste treatment, and food production were decline during 1982 to 2008, while other functions were improved. The rank of the contribution by each ecosystem service function was also estimated. It was basically stable except for relatively obvious decline in the rank of waste treatment and food production. In 2008, the rank order for each ecosystem service was as follows from high to low, soil formation and retention, biodiversity protection, water supply, climate regulation, gas regulation, waste treatment, raw material, recreation and culture, and food production. Soil formation and retention was the highest during the study period.

**Table 5:** Values of different ecosystem service functions in 1982, 2002, and 2008.

	1982			2002			2008		
	ESVs (10^6^ US$·yr^−1^)	%	Rank	ESVs (10^6^ US$·yr^−1^)	%	Rank	ESVs (10^6^ US$·yr^−1^)	%	Rank
Gas regulation	0.678	10.73	6	0.826	11.26	6	1.529	13.56	5
Climate regulation	0.791	12.53	5	0.936	12.75	5	1.540	13.65	4
Water supply	0.800	12.67	4	0.960	13.09	4	1.610	14.28	3
Soil formation and retention	1.141	18.06	1	1.260	17.17	1	1.764	15.65	1
Waste treatment	0.938	14.85	2	1.015	13.84	3	1.078	9.56	6
Biodiversity protection	0.915	14.49	3	1.054	14.37	2	1.738	15.42	2
Food production	0.466	7.38	7	0.506	6.90	7	0.367	3.25	9
Raw material	0.247	3.91	9	0.390	5.32	8	0.881	7.81	7
Recreation and culture	0.339	5.37	8	0.388	5.29	9	0.768	6.81	8
Total	6.315	100.00		7.336	100.00		11.275	100.00	

### 3.3 Spatial distribution of ESVs

**Figure 5:**
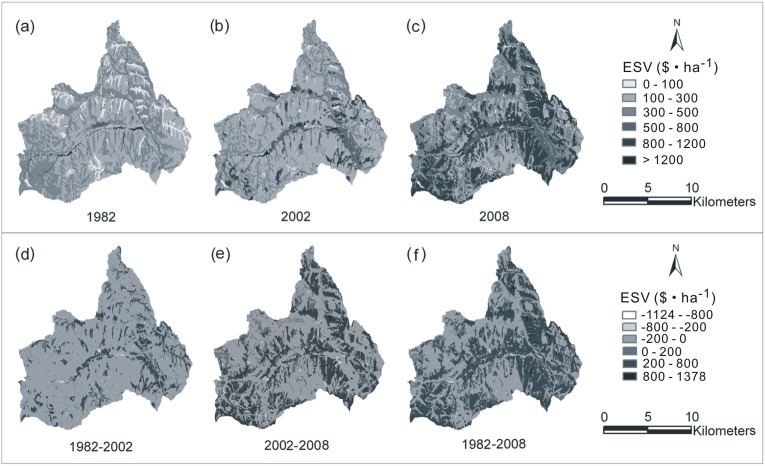
Spatial and temporal distribution of ecosystem service values (ESVs) in Hechuan Town from 1982 to 2008. The spatial distribution of ESVs in 1982 (a), 2002 (b) and 2008 (c), and the spatial-temporal changes of ESVs between time intervals from 1982 to 2002 (d), 2002 to 2008 (e) and 1982 to 2008 (f).

Maps of ESVs in different periods (Fig. 5) showed the spatial distribution of ESVs of unit area in Hechuan town, directly reflecting the difference of ESVs among land use types. In 1982, the ESVs>4000 mostly appeared in the center of the town where river and river terrace located. It was because water body and orchard which intensely distributed in river terrace for its high water demand both had high ESVs. Therefore, due to the orchard increasing intensely and the forest increasing scatteredly, the increase of ESVs also mainly happened across the river terrace in 2002. Since 2008, the ESVs>4000 spread widely with the increase of forestland transformed from cropland. The lowest ESVs mostly occurred in the gully where unused land was distributed in 1982. With vegetation recovery in the gully, the low ESVs happened from gully to terraced hillside where cropland with low ESVs was distributed in 2008. Fig. 5d–f showed the temporal change of ESVs spatial distribution. The change characteristic of 2002 to 2008 was adjacent to that during the total study period, reflecting that the change of ESVs mainly occurred after 2002, just after the Grain to Green project.

### 3.4 Relationship between ESVs and land use pattern

From the above analysis on the change of land use and ESVs in quantity and spatial distribution, we could infer there was some relationship between land use change and ecosystem service. To quantitively understand the relationship, the correlation analysis and regression analysis between ESVs and landscape pattern metrics was conducted.

Table 6 showed there existed significant correlations between ESVs and many landscape metrics (p<0.01), which explained that landscape pattern affected ESVs significantly. For example, the correlation coefficients between total ESVs and landscape metrics showed that there existed significantly positive relationship between SHDI (0.433), PLAND_3 (0.677), SHAPE_AM_3 (0.744), IJI_4 (0.513) and ESVs, and negative relationship between LUII (−0.634), PLAND_1 (−0.752) and ESVs. It reflected that the diversity and intensity of land use had important effects on total ESVs. It also reflected that cropland, forestland and grassland were the land use types which had significant effects on total ESVs. On quantity,the less the cropland and the more the forestland, the higher the total ESVs were. As to the landscape shape, the more regular the cropland and the more complex the forestland, the higher the total ESVs were. The higher the IJI of grassland, the higher the total ESVs were. This indicated that the connectivity of grassland was important for ecosystem service.

**Table 6:** Correlation coefficients between ecosystem service values (ESVs) and landscape pattern metrics.

	TESVs	ESVs_1	ESVs_2	ESVs_3	ESVs_4	ESVs_5	ESVs_6	ESVs_7	ESVs_8	ESVs_9
PD	0.035	0.497*	0.509*	0.539*	0.477*	0.516*	0.499*	−0.221	0.547	0.478*
SHAPE_AM	0.326	−0.216	−0.220	−0.188	−0.177	−0.088	−0.205	0.026	−0.290	−0.166
IJI	0.292	0.624*	0.635*	0.639*	0.597*	0.534*	0.621*	−0.293	0.687	0.586*
SHDI	0.433*	0.763*	0.764*	0.766*	0.741*	0.507*	0.765*	−0.636*	0.775*	0.765*
LUII	−0.634*	−0.681*	−0.658*	−0.618*	−0.675*	−0.113	−0.684*	0.977*	−0.599*	−0.734*
PLAND_1	−0.752*	−0.810*	−0.795*	−0.772*	−0.811*	−0.334	−0.815*	0.952*	−0.742*	−0.853*
PD_1	0.045	−0.055	−0.063	−0.056	−0.054	−0.113	−0.051	−0.134	−0.091	−0.022
SHAPE_AM_1	−0.476*	−0.369	−0.358	−0.330	−0.368	−0.063	−0.369	0.495	−0.328	−0.390
IJI_1	0.189	0.542*	0.552*	0.530*	0.510*	0.405	0.533*	−0.199	0.619	0.485*
PLAND_2	0.323	0.527*	0.541*	0.590*	0.520*	0.595*	0.534*	−0.246	0.558	0.525*
PD_2	0.420	0.457*	0.471*	0.515*	0.449	0.529*	0.463*	−0.193	0.491	0.452
SHAPE_AM_2	0.159	0.450	0.468*	0.541*	0.439	0.629*	0.460*	−0.149	0.493	0.452
IJI_2	0.207	0.392	0.409	0.483*	0.376	0.583*	0.402	−0.115	0.438	0.396
PLAND_3	0.677*	0.984*	0.983*	0.941*	0.975	0.558*	0.980*	−0.770*	0.988*	0.961*
PD_3	0.276	0.631*	0.637*	0.629	0.625	0.477*	0.629*	−0.383	0.653	0.606*
SHAPE_AM_3	0.744*	0.828*	0.827*	0.780*	0.820	0.449*	0.821*	−0.623*	0.836*	0.799*
IJI_3	0.040	0.231	0.241	0.291	0.208	0.356	0.236	−0.069	0.276	0.231
PLAND_4	0.224	−0.192	−0.212	−0.203	−0.159	−0.298	−0.181	−0.264	−0.311	−0.110
PD_4	−0.294	0.199	0.201	0.190	0.160	0.101	0.192	−0.086	0.257	0.171
SHAPE_AM_4	0.455	−0.061	−0.065	−0.049	−0.028	−0.029	−0.053	−0.086	−0.125	−0.022
IJI_4	0.513*	0.717*	0.719*	0.705*	0.697*	0.457*	0.715*	−0.539*	0.739*	0.701*
PLAND_5	−0.035	0.290	0.313	0.381	0.279	0.578*	0.297	0.081	0.357	0.271
PD_5	−0.047	0.244	0.269	0.322	0.244	0.548*	0.248	0.188	0.314	0.208
SHAPE_AM_5	0.118	0.081	0.082	0.053	0.072	−0.009	0.075	−0.004	0.105	0.055
IJI_5	0.307	0.461*	0.470*	0.525*	0.446*	0.512*	0.471*	−0.312	0.482	0.477*
PLAND_6	0.047	0.139	0.167	0.360	0.148	0.852	0.170	0.064	0.153	0.207
PD_6	−0.160	0.122	0.137	0.198	0.105	0.371	0.128	0.080	0.172	0.118
SHAPE_AM_6	0.378	0.088	0.086	0.140	0.080	0.141	0.099	−0.218	0.067	0.137
IJI_6	0.020	0.201	0.217	0.276	0.197	0.438	0.208	0.028	0.239	0.197
PLAND_7	−0.385	−0.447	−0.474*	−0.525*	−0.507*	−0.769*	−0.456*	−0.041	−0.442	−0.422
PD_7	−0.270	−0.105	−0.129	−0.182	−0.154	−0.494*	−0.114	−0.245	−0.108	−0.089
SHAPE_AM_7	−0.236	−0.313	−0.329	−0.434	−0.348	−0.650*	−0.333	0.150	−0.279	−0.358
IJI_7	0.210	0.416	0.408	0.287	0.402	−0.083	0.394	−0.254	0.443	0.341

TESVs: the total ecosystem service values (ESVs); ESVs_1: the ESVs of gas regulation; ESVs_2 climate regulation; ESVs_3: the ESVs of water supply; ESVs_4: the ESVs of soil formation and retention; ESVs_5: the ESVs of waste treatment, ESVs_6: the ESVs of biodiversity protection; ESVs_7 the ESVs of food production; ESVs_8: the ESVs of raw material; ESVs_9: the ESVs of recreation and culture.

PD: patch density; SHAPE_AM: area-weighted mean shape index; IJI: Interspersion and Justaposition Index; SHDI: Shannon’s diversity index; LUII: land use intensity index; PLAND: percentage of landscape. The 1, 2, 3, 4, 5, 6, 7 after the above landscape metrics respects different landscape, that is cropland, orchard, forestland, grassland, residential land, water body and unused land, respectively.

*significant at 0.01 level.

Correlation also occurred between ESVs of all the functions and landscape metrics (Table 6). However, the relationships between ESVs of different functions and landscape pattern were different. For example, the correlation between food production and landscape pattern was almost opposite from that between other ecosystem functions and landscape pattern. For example, PLAND_1 had a positive effect on food production; SHDI, PLAND_3, SHAPE_3, and IJI_4 had a negative effect on food production. It could infer that there were contradictions between food production and other ecosystem functions.

As shown in Table 7, the result of regression analysis further explained that the ESVs was correlated significantly with landscape pattern. The total ESVs could be predicted by PLAND on cropland, SHAPE_AM on grassland, and SHDI. ESVs of all kinds of ecosystem functions also could be explained by landscape metrics. These regression equations indicated that landscape-level metrics (such as SHDI and LUII) and class_level metrics (such as PLAND of forestland, orchard, and cropland, unused land, SHAPE of forestland, IJI of cropland) acted as predictors for categories of ecosystem services. Specifically, the proportion of forest (PLAND_3) accounted for almost all of the categories of ecosystem services.

**Table 7:** Regression analysis between ecosystem service values (ESVs) and landscape patterns (n = 36).

Dependent	Standardized coefficients regression	R^2^	Sig.
Gas regulation	0.878×PLAND_3+0.166×PLAND_2-0.099×PLAND_1-0.068×IJI_1	0.990	*
Climate regulation	0.790×PLAND_3-0.197×PLAND_7-0.190×LUII+0.081×PLAND_2	0.998	*
Water supply	0.665×PLAND_3-0.317×PLAND_7-0.254×LUII+0.106×PLAND_2	0.955	*
Soil formation and retention	0.684×PLAND_3-0.301×PLAND_7-0.284×LUII+0.066×PLAND_2	0.998	*
Waste treatment	0.672×PLAND_6+0.365×PLAND_3-0.352×PLAND_7+0.051×PLAND_2+0.049×PLAND_5	0.993	*
Biodiversity protection	0.059×SHDI +0.861×PLAND_3-0.033×SHAPE_AM_3+0.133×PLAND_1	0.967	*
Food production	0.742×LUII-0.173×PLAND_3-0.052×SHDI+0.106×PLAND_1	0.991	*
Raw material	0.964×PLAND_3+0.091×SHDI+0.068×LUII	0.981	*
Recreation and culture	−0.747×PLAND_1+0.380×IJI_1	0.853	*
Total	-0.588×PLAND_1+0.569× SHAPE_AM_3-0.303×SHDI	0.709	*

*significant at 0.01 level.

PLAND_1: the percentage of cropland; PLAND_2: the percentage of orchard; PLAND_3: the percentage of forestland; PLAND_5: the percentage of residential land; PLAND_6: the percentage of water body; PLAND_7: the percentage of unused land; SHAPE_AM_3: the area-weighted mean shape index of forestland; IJI_1: the Interspersion and Justaposition Index of cropland; LUII: land use intensity index; SHDI: Shannon’s diversity index.

## Discussion

### 4.1 Reliability of ESVs

This study estimated ESVs by multiplying the area for each land use types by the corresponding value coefficients. As discussed in the previous researches, estimations using this method was coarse with high variation and uncertainty for the following reasons, limitations on the economic evaluation [1], problems of double counting and scales [40], [41], [42], the complex, dynamic and nonlinear ecosystems [43], the imperfect matches of land use categories as proxies [38] and the accuracy of the ecosystem value coefficients [5]. This study also existed such uncertainty on ESVs estimation. For example, the value coefficient of orchard, determined by the average of forest and grassland, was an approximate estimation and need a further exploration. However, the estimation of temporal variation on ESVs was considered to be more reliable than that of cross-sectional analysis [5]. In addition, the sensitivity analysis of the estimated ESVs with 50% adjustment in the value coefficients was conducted. The result showed that the sensitivity coefficients of all land use categories were lower than 1 (Table 8), which suggested that despite of the above limitations, the estimated ESVs are reliable and useful for subsequent study.

**Table 8:** The coefficient of sensitivity (CS) resulting from adjustment of ecosystem valuation coefficients.

	Cropland	Orchard	Forestland	Grass land	Water body	Unused land
1982	−0.430	−0.012	−0.008	−0.500	−0.039	−0.011
2002	−0.428	−0.052	−0.169	−0.309	−0.041	−0.002
2008	−0.129	−0.048	−0.574	−0.223	−0.027	−0.001

### 4.2 Relationship between ESVs and landscape pattern

It is usually assumed that land use can affect the ecosystem service. Moreover, a few studies showed that there was a correlation between landscape pattern and ESVs [41], [44]. This study signified this statement at town scale on the Loess Plateau. Land use configuration, land use intensity, landscape diversity, fragmentation and connectivity all affected ecosystem service.

The correlation analysis between ESVs and PLAND implied land use structure had significant impact on ecosystem service. Especially, the increase of forestland and the decrease of cropland played an important part in improving the ESVs in the past twenty years. It is closely related to the Grain to Green project comprehensively started in study area since 2002. In the project, measures for optimizing land use structure were implemented, including restoring slope cropland into forest and grassland, banning grazing, transforming slopes into terraces, and building reservoirs, etc. Forestland and grassland increased by 423.19% (27.54% of the study area) and 10.93% (3.15% of the study area), and cropland decreased by 53.59%(31.49% of the study area) (Table 3). The increase of ESVs due to the increase of forestland occupied 46.28% of the total ESVs in 2008 (Table 4). The result of the correlation analysis between ESVs and PLAND reflected that vegetation recovery could strongly enhance ecosystem service, and it was coincident with many other studies on the Loess Plateau [17], [18], [23], [45]. LUII, which also related to the proportion of land use types, implied the intensity of human activities. This study showed land use intensity had a negative effect on ecosystem service with negative correlation coefficients (−0.634) (Table 6). It was coincident with some studies on ESVs change under urbanization [5], [31]. These studies showed that urbanization which means the increase of land use intensity led to considerable declines in ESVs.

Landscape diversity always presents high positive relevance with biodiversity [46]. Our results were coincident to previous statements given the positive relationships between SHDI and biodiversity conservation. However, there were studies reporting the negative relationships between them, in which the increase of SHDI was the result of rapid urban sprawl [31]. In our study, the increase of SHDI was because land use structure became more balanced, which was the result of the increase of forestland. In addition, landscape diversity could also promote agricultural production [47]. Our study disagreed with this statement, and showed that food production was weakened with landscape diversification. It was because that the increase of SHDI was the result of a larger number of conversion from cropland to forestland. Therefore, the relationship between landscape diversity and biodiversity conservation as well as food production should not be treat as the same but be understood considering the driving force of SHDI change.

Fragmentation could lead to declining habitat quality, lower wildlife survival, and limited movement of soil microorganisms [48], and subsequently cause the decrease of ecosystem service [30]. Our study disagreed with this statement. For example, PD of the total landscape, PD_Forest, PD_orchard and shape_ Forest revealed significantly positive impacts on most categories of ESVs (Table 6–7). The increase of PD and the decrease of connectivity of landscape were usually simultaneous, which is disagreed in our study (Fig. 3 and Fig. 4). The landscape became more contiguous as IJI shown. Table 6 showed the IJI had significantly positive impacts on ESVs. Especially, the increase of IJI of grassland promoted the total ESVs and all categories of ESVs. This maybe because the connectivity of landscape has contribution to habitat corridors [49] and forest production [50].

Based on the relationship between ESVs and landscape pattern, we could improve the ecosystem service by the adjustment of land use policy. On the one hand, continuing to implement the Grain to Green project is helpful for improving ESVS, because it could increase the vegetation coverage, decline the intensity of land use, and make cropland become regular by canceling the slope cropland. On the other hand, diversified agriculture gathering planing fruit trees, planting crops and breeding, which could promote the diversification of land use, should be encouraged to increase both ESVs and farmer’s incomes.

## Conclusion

ESVs at town scale in the Loess Plateau were estimated in Hechuan town of Ningxia Hui Autonomous Region from 1982 to 2008. It was concluded that ESVs varied with land use change. ESVs in 1982, 2002, and 2008 were US$ 6.315, US$ 7.336 and US$ 11.275 million respectively. Among all the land use types, forestland, grassland and cropland had important contribution (>90%) on ESVs. The total ESVs increased slowly by 16.17% due to the decrease of grassland from 1982 to 2002, while the total ESVS increased significantly by 67.61% due to the increase of forestland from 2002 to 2008. Areas with high services level were mainly located in the center due to orchard and east due to forestland, while areas with low services level mainly located in the north and south sides due to cropland.

Land use pattern had a significant effect on ecosystem service in our study by analyzing and discussing the relationship between landscape pattern and ESVs. The proportion of forestland had a positive effect on ecosystem service while that of cropland had a negative effect on ESVs. The diversity and interspersion of landscape both had a positive effect on ESVs. Land use intensity which reflects the intensity of human activities had a negative effect on ESVs. Fragmentation had positive effect on ESVs, which was disagreed with the previous studies because the fragmentation in study area was related to the increased patch of such land use types as forestland, water body, orchard.

Based on the results of this study, it was conclude that land use pattern was important for ecosystem service. Therefore, we could improve the ecosystem service by the adjustment of land use policy. Continuing the Grain to Green project is reasonable and significant because it could increase the vegetation coverage and decline land use intensity. Diversified agriculture collecting planing fruit trees, growing food and breeding should be encouraged, because it could not only promote ecosystem service by increasing landscape diversification but also improve people’s incomes.
